# Conditional Deletion of Activating Rearranged During Transfection Receptor Tyrosine Kinase Leads to Impairment of Photoreceptor Ribbon Synapses and Disrupted Visual Function in Mice

**DOI:** 10.3389/fnins.2021.728905

**Published:** 2021-11-05

**Authors:** Wei-Hao Peng, Meng-Lin Liao, Wan-Chun Huang, Pei-Kang Liu, Sarah R. Levi, Yun-Ju Tseng, Chia-Ying Lee, Lung-Kun Yeh, Kuan-Jen Chen, Chung-Liang Chien, Nan-Kai Wang

**Affiliations:** ^1^School of Medicine for International Students, College of Medicine, I-Shou University, Kaohsiung, Taiwan; ^2^Department of Anatomy and Cell Biology, College of Medicine, National Taiwan University, Taipei, Taiwan; ^3^Department of Ophthalmology, Kaohsiung Medical University Hospital, Kaohsiung Medical University, Kaohsiung, Taiwan; ^4^School of Medicine, College of Medicine, Kaohsiung Medical University, Kaohsiung, Taiwan; ^5^Institute of Biomedical Sciences, National Sun Yat-sen University, Kaohsiung, Taiwan; ^6^Department of Ophthalmology, Edward S. Harkness Eye Institute, Columbia University Irving Medical Center, Columbia University, New York, NY, United States; ^7^Department of Ophthalmology, Chang Gung Memorial Hospital, Taoyuan, Taiwan; ^8^College of Medicine, Chang Gung University, Taoyuan, Taiwan

**Keywords:** GDNF family of ligands (GFL), rearranged during transfection (RET), mouse retina, ribbon synapses, Cre-loxP knockout mice

## Abstract

**Purpose:** The rearranged during transfection (RET) receptor tyrosine kinase plays a key role in transducing signals related to cell growth and differentiation. *Ret* mutant mice show abnormal retinal activity and abnormal levels and morphology of bipolar cells, yet die on the 21^st^ day after birth as a result of renal underdevelopment. To extend the observation period, we generated the *Ret* conditional knockout *Chx10-Cre;C-Ret^*lx/lx*^* mouse model and analyzed the retinal function and morphological changes in mature and aging *Chx10-Cre;C-Ret^*lx/lx*^* mice.

**Methods:** Retina-specific depletion of *Ret* was achieved using mice with floxed alleles of the *Ret* gene with CHX10-driven Cre recombinase; floxed mice without Cre expression were used as controls. Retinal function was examined using electroretinography (ERG), and 2-, 4-, 12-, and 24-month-old mice were analyzed by hematoxylin staining and immunohistochemistry to evaluate retinal morphological alterations. The ultrastructure of photoreceptor synapses was evaluated using electron microscopy.

**Results:** The results of the ERG testing showed that b-wave amplitudes were reduced in *Chx10-Cre;C-Ret^*lx/lx*^* mice, whereas a-waves were not affected. A histopathological analysis revealed a thinner and disorganized outer plexiform layer at the ages of 12 and 24 months in *Chx10-Cre;C-Ret^*lx/lx*^* mice. Moreover, the data provided by immunohistochemistry showed defects in the synapses of photoreceptor cells. This result was confirmed at the ultrastructural level, thus supporting the participation of *Ret* in the morphological changes of the synaptic ribbon.

**Conclusion:** Our results provide evidence of the role of *Ret* in maintaining the function of the retina, which was essential for preserving the structure of the synaptic ribbon and supporting the integrity of the outer plexiform layer.

## Introduction

The retina receives light signals at levels that span several orders of magnitude. Adaptive changes to different light levels occur at multiple sites within the retinal signal transmission and act together in processing the light information. In contrast to conventional neurons, photoreceptors do not signal via action potentials; rather, they continuously translate light into a graded transmitter release, with the highest exocytosis rates in the dark. To accomplish this task, photoreceptors and retinal bipolar cells contain a specialized type of synapse, the so-called ribbon synapse. The ribbon synapse is a specialized synaptic structure located in the outer plexiform layer (OPL) of the retina in which a synaptic “triad” is formed between the axonal pedicles of rods and cones and the dendrites of horizontal and bipolar cells. Through this special synapse, visual signals are transmitted from photoreceptors to bipolar and horizontal cells (Heidelberger et al., 2005). Morphologically, ribbon synapses are anchored to the plasma membrane in close vicinity to voltage-gated Ca^2+^ channels and are typically surrounded by a large number of synaptic vesicles (Sterling and Matthews, 2005). A previous study showed that the lack of active-zone-anchored synaptic ribbons reduced the presynaptic readily releasable vesicle pool and impaired synchronous visual signaling, thus affecting visual function (LoGiudice and Matthews, 2009). Therefore, photoreceptor ribbon synapses play an important role in visual function.

To protect central nervous system (CNS) cells, mutation-independent neuroprotective strategies—such as the glial-cell-line-derived neurotrophic factor (GDNF), the brain-derived neurotrophic factor (BDNF), the nerve growth factor (NGF), and the ciliary neurotrophic factor (CNTF)—have been applied to, and their therapeutic potential has been demonstrated in, the management of retinal impairment in various animal models (Chinskey et al., 2014). GDNF is a distant member of the transforming growth factor β (TGF-β) superfamily and a founder protein of the GDNF-family ligands (GFLs), which include neurturin (NRTN), artemin (ARTN), and persephin (PSPN) (Airaksinen and Saarma, 2002). All four GFLs (GDNF, NRTN, ARTN, and PSPN) signal via the activation of the rearranged during transfection (RET) receptor tyrosine kinase, a single-pass transmembrane protein that contains four cadherin-like repeats in the extracellular domain and a typical intracellular tyrosine kinase domain (Durbec et al., 1996). These GFLs promote the survival of various neurons, including peripheral neurons and central motor and dopamine neurons, and have been suggested as candidate therapeutic agents for neurodegenerative diseases (Takahashi, 2001).

The function of GDNF in the nervous system has been investigated in many studies. GDNF promotes the differentiation and survival of rat midbrain dopamine neurons and increases the outgrowth of neurites and dopamine uptake *in vitro* (Lin et al., 1993). Moreover, GDNF stimulates the formation of new axon terminals in dopamine neurons, which has led to an increased interest in the therapeutic potential of GDNF for the management of Parkinson’s disease (Bourque and Trudeau, 2000). In addition, a previous study showed that GDNF supports the survival of spinal motoneurons (Henderson et al., 1994). In the eye, GDNF is mainly expressed in the retina and has potential therapeutic value by providing neuroprotection in the context of retinal degeneration (Koeberle and Ball, 1998). Moreover, GDNF was reported to be able to rescue retinal ganglion cells after axotomy (Yan et al., 1999) and to be very effective in retarding photoreceptor degeneration in the retinal degeneration 1 (*rd1*) mouse model (Frasson et al., 1999). Subretinal injection of GDNF decreased the loss of photoreceptors and provided a significant functional rescue, as demonstrated by recordable electroretinography (ERG) (Frasson et al., 1999). These studies suggest that GDNF-mediated RET signaling affects retinal function.

GDNF was shown to be a RET ligand, and extensive studies of intracellular signaling through RET have been performed. Specifically, mice carrying loss-of-function mutations in a variety of GFLs or in their receptors exhibited either a loss of sensory neuron populations or a loss of specific types of neurons (Airaksinen and Saarma, 2002; Ernsberger, 2008). Moreover, GDNF/RET signaling plays crucial roles in renal development (Costantini and Shakya, 2006) and the regulation of spermatogonia differentiation (Meng et al., 2000). In addition, RET mutations have been found to cause several human diseases, such as papillary thyroid carcinoma, multiple endocrine neoplasia types 2A and 2B, and Hirschsprung’s disease. *Ret*-knockout mice exhibit a lack of enteric neurons and superior cervical ganglia, as well as renal agenesis or dysgenesis (Schuchardt et al., 1994; Moore et al., 1996). Furthermore, a previous study demonstrated that abnormal retinal activity in NRTN- or *Ret*-deficient mice was associated with abnormal process extension of horizontal cells and bipolar cells into the outer nuclear layer (ONL), as well as a severely disrupted OPL with very sparse dendrites and axons of horizontal cells (Brantley et al., 2008). These results suggest that RET signaling is involved in retinal development. However, *Ret*-deficient mice die before postnatal day 21; thus, further evaluation of their retinal phenotype in adulthood is lacking in the literature (Schuchardt et al., 1994).

The Cre-loxP system is widely used as a powerful genetic tool for generating conditional knockout mice. Researchers can use this system to investigate genes of interest in a tissue/cell- (spatial control) and/or time- (temporal control) specific manner when straight knockout of the genes of interest causes embryonic lethality. In this study, we aimed to investigate the function of *Ret* in the retina by generating conditional knockout mice using the Cre-loxP system. Although conditional *Ret^*RETfloxEGFP/RETfloxEGFP*^:Six3* Cre knockout mice have been reported, the long-term effect of this intervention on the retina remains unknown (Brantley et al., 2008). To overcome this limitation, we deleted *Ret* exclusively in the retina by crossing homozygous *Ret* conditional knockout mice (i.e., *Ret*^*lx/lx*^ mice) with mice expressing Cre recombinase under the control of the *Chx10* gene. The *Chx10* gene is specifically expressed in the retinal progenitor cells at the early stage of eye development (E11.5), followed by a restricted expression in the bipolar cells as the progenitor cells differentiate and exit the cell cycle (Rowan and Cepko, 2004). We analyzed the retinal network in mature and aged *Chx10-Cre;C-Ret^*lx/lx*^* mice and investigated the alteration in the ultrastructure of synaptic ribbons in *Chx10-Cre;C-Ret^*lx/lx*^* mice specifically.

## Materials and Methods

### Animals

**FIGURE 1:**
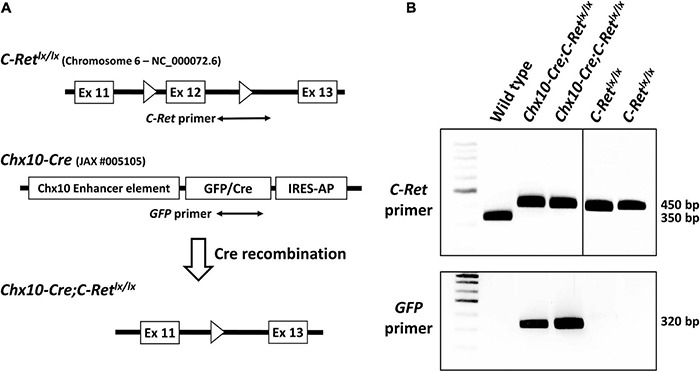
Targeting strategy for the generation of conditional *Ret*-knockout mice and genotyping of *C-Ret^*lx/lx*^* and *Chx10-Cre;C-Ret^*lx/lx*^* mice using polymerase chain reaction (PCR). The Cre-loxP system was used to generate conditional *Ret*-knockout mice. In *C-Ret^*lx/lx*^* mice, the target, exon 12, of the *C-Ret* gene is flanked by two loxP sites (**A**, top). The Chx10-Cre mouse model (JAX #005105) was generated by Chx10 BAC transgenes using a GFP/Cre translational fusion protein combined with internal ribosome entry sequence-human placental alkaline phosphatase cassette (IRES-AP) under the control of Chx10 enhancer elements (**A**, middle). In *Chx10-Cre;C-Ret^*lx/lx*^* mice, Cre recognizes the loxP sites and excises the target exon 12 together with one loxP sequence while recombining the two ends of the remaining sequences, thus causing the permanent deletion of the target *C-Ret* gene. Exons are depicted as white boxes and *loxP* sites are shown as white triangles (**A**, bottom). A PCR analysis of all three genotypes (wild-type, *C-Ret^*lx/lx*^*, and *Chx10-Cre;C-Ret^*lx/lx*^*) using the *C-Ret* primers (**A**, top) showed that a 350 bp signal was detected in the wild-type (WT) mice and 450 bp signals were observed in *C-Ret^*lx/lx*^* and *Chx10-Cre;C-Ret^*lx/lx*^* mice (**B**, top). The PCR amplification of *GFP* (**A**, middle), which was detected as a 320 bp fragment, was observed exclusively in *Chx10-Cre;C-Ret^*lx/lx*^* mice (**B**, bottom).

All animal procedures were performed according to the guidelines of the Association for Research in Vision and Ophthalmology Statement for the Use of Animals in Ophthalmic and Vision Research and were approved by the Institutional Animal Care and Use Committee of the National Taiwan University. *Ret^*Ret* < tm1.1Kln >^* mice were generated by Dr. Klein (Kramer et al., 2007) by targeting a construct encompassing exons 11–13 of the *Ret* gene with Lox P sites flanking exon 12 (Figure 1A) (termed *C-Ret^*lx/lx*^* mice hereafter). The generation of conditional knockout mice with *Ret* gene deletion in the retina was achieved by crossing *C-Ret^*lx/lx*^* mice carrying LoxP sites flanking exon 12 of the *Ret* gene (Kramer et al., 2006) with mice expressing *Chx10-Cre* specifically in the retina (JAX: Stock No. 005105). All mice were housed in groups of four to five animals per cage in a room that was kept at 23 ± 1°C and 55% ± 5% humidity with a 12-h light/dark cycle, and were given *ad libitum* access to food and water. All mice used in our experiments were genotyped to confirm the absence of the *rd8* and *rd1* mutations as they may be present in vendor lines and subsequently confound ocular-induced mutant phenotypes (Errijgers et al., 2007; Mattapallil et al., 2012).

### Mouse Genotyping

Mice were genotyped and verified using polymerase chain reaction (PCR) analysis. For genotyping, genomic DNA was isolated from a section of mouse tail, optic nerve and retina using an Aquadien^TM^ kit (Bio-Rad, Richmond, CA, United States) according to the manufacturer’s instructions. Mice homozygous for *C-Ret^*lx/lx*^* were identified using the C-*Ret* forward (5′-*CCA ACA GTA GCC TCT GTG TAA CCC C*-3′) and reverse (5′-*GCA GTC TCT CCA TGG ACA TGG TAG CC*-3′) primers span the loxP in intron 12 (Figure 1A, top). Optic atrophy type 1 *(Opa1)* forward (5′-*GAG CTG AGA GGG AGT GAA GAG AGG*-3′) and reverse (5′-*CCC AAA ACT CCT TTA TCC CAG TGA C*-3′) primers could serve as the positive control. Furthermore, the *Chx10-Cre* mice carried *EGFP* fused with Cre recombinase; therefore, primers that amplify *EGFP* were also used to detect the presence of Cre recombinase. The thermal cycling conditions consisted of 30 cycles of 30 s at 94°C, 30 s at 55°C, and 50 s at 72°C. Reactions contained 200 ng of template DNA, 0.5 μM primers, 100 μM dNTPs, 9% glycerol, 2.5 U of *Taq* polymerase, 1.8 mM MgCl_2_, and 1× PCR buffer (GIBCO BRL) in a volume of 20 μL. The PCR products were resolved via 2% agarose gel electrophoresis using Gel Red (Invitrogen/Life Technologies) as the visualizing dye. The DNA bands were visualized using a ChemiDoc Imaging System (Bio-Rad).

### Electroretinography

Electroretinography was performed as described previously (Wang et al., 2010). After 12 h of adaptation in the dark, the mice were prepared for the ERG recordings using an Espion ERG System (Diagnosys LLC; Lowell, MA, United States) under dim red light. The animals were kept on a heating pad (Mycoal, Tochigi, Japan) during the ERG recordings, to maintain a constant body temperature. Mice were anesthetized via intraperitoneal injection of 0.1 mL of a mixed solution (1 mL of ketamine at 100 mg/mL and 0.1 mL of xylazine at 20 mg/mL in 8.9 mL of PBS) per 10 g of body weight, and pupils were dilated with topical 2.5% phenylephrine hydrochloride and 1% tropicamide. The test protocol consisted of 11 dark-adapted and nine light-adapted steps. The light intensities of the stimuli used for scotopic serial intensity ERG were –3.6, –3.2, –2.8, –2.4, –2.0, –1.6, –1.2, –0.6, 0.0, 0.4, and 0.9 log cd.s/m^2^ in sequence. The intervals between each stimulus varied from 2 to 30 s, and the number of repeats varied from 10 to 4 times. After the completion of dark-adapted recordings, the animals were exposed to a full-field 30 cd/m^2^ white background for 10 min; subsequent steps were delivered on top of this continuous background. The single-flash stimuli applied after light adaptation consisted of −0.1, 0.1, 0.3, 0.8, 1.0, 1.2, and 1.47 log cd.s/m^2^. The intervals between each stimulus varied from 1 to 10 s, and the number of repeats varied from 3 to 10 times. A digital band-pass filter ranging from 0.3 to 300 Hz was used to isolate signals after the waves were recorded. The a-wave amplitude was measured from the baseline to the trough of the a-wave, and the b-wave was measured from the trough of the a-wave to the peak of the b-wave.

### Tissue Preparation

Mice were sacrificed at 2, 4, 12, and 24 months of age and their eyes were enucleated and fixed in 4% paraformaldehyde (PFA) in PBS for 1 h at room temperature (RT). For a better retinal infiltration of 4% PFA, corneas were partially removed and then placed in 4% PFA at 4°C for ∼1 h. After washing in PBS three times, the tissues were incubated in a 30% sucrose solution in PBS at 4°C for 3 days. Tissues were embedded in optimum cutting temperature (OCT) compound (Thermo, Pittsburgh, PA, United States), snap frozen in liquid nitrogen, and immediately stored at –80°C. Cryosections (14 μm in thickness) were cut and collected on slides (Matsunami, Osaka, Japan). All slides were stored at –80°C before use. Before any staining process, the slides were air dried for 15 min and washed in PBS.

### Hematoxylin Staining

Sections were stained with hematoxylin for 1 min, then washed with running water for 5 min. The stained sections were mounted with an aqueous mounting medium (EMS, Hatfield, PA, United States) and viewed under a microscope (Olympus CH-2 system, Tokyo, Japan). The brightness and contrast of photomicrographs were adjusted for maximum clarity using Adobe Photoshop CS5 (Adobe Systems, San Jose, CA, United States). The retinas of three mice in each group underwent further histological analysis.

### Immunohistochemistry

Sections were blocked with blocking buffer [5% fetal bovine serum (FBS) in PBS containing 0.1% Triton X-100] for 1 h at RT, followed by incubation with the primary antibodies (Table 1) diluted in 3% FBS in PBS at 4°C overnight. The sections were subsequently incubated with the secondary antibodies for 1 h at RT. After washing three times in PBS, they were mounted with mounting medium (EMS, Hatfield, PA, United States) and viewed under a Leica DM6000 Confocal Fluorescence Imaging Microscope (Leica Microsystems, Wetzlar, Germany).

**TABLE 1:** List of antibodies applied for immunohistochemistry.

**Antigen**	**Antiserum**	**Cell type**	**Source**	**Catalog**	**Dilution factor**
α-internexin	Mouse anti-internexin, α, C-terminus, clone 2E3	Horizontal cell and ganglion cell	Millipore	MAB5224	1:100
PKC-α	Mouse anti-PKC-α (H-7)	Rod bipolar cell	Santa Cruz Biotechnology	Sc-8393	1:100
PKC-α	Rabbit anti-PKC-α (H-300)	Rod bipolar cell	Santa Cruz Biotechnology	Sc-10800	1:100
Synaptophysin	Rabbit anti-synaptophysin	Photoreceptor	Abcam	Ab-14692	1:100
Calbindin	Rabbit anti-calbindin	Horizontal cell	Invitrogen	PA1-931	1:500
PSD-95	Mouse anti-PSD95	Photoreceptor	NeuroMab	75-028	1:500
EGFP	Rabbit anti-(GFP)	GFP expressing cells	Millipore	Ab-3080	1:500
Mouse IgG	Goat anti-mouse IgG Alexa 488 and 594		Invitrogen	A11001 A11005	1:200
Rabbit IgG	Goat anti-rabbit IgG Alexa 488 and 594		Invitrogen	A11008 A11012	1:200

### Transmission Electron Microscopy and Quantification

Retinas were isolated and fixed in 2% glutaraldehyde and 2% PFA in 0.1 M PB at 4°C overnight. After postfixing in 1% osmium tetroxide for 1 h, tissue samples were dehydrated in a graded ethanol series and embedded in epoxy resin (EMS, Hatfield, PA, United States). Ultrathin sections (70 nm in thickness) were collected on copper grids and stained with uranyl acetate and lead citrate before examination under a Hitachi H-7100 electron microscope (Hitachi, Tokyo, Japan) equipped with a Gatan 832 digital camera (Gatan, Inc., Pleasanton, CA, United States).

For the quantification of synaptic ribbon conditions, images of the OPL were taken at a magnification of 40,000X. Approximately 500 photoreceptor terminals for each age were examined and classified into different categories of rod-shaped ribbon profiles.

### Histological Quantification

The thickness of the ONL and inner nuclear layer (INL) was quantified from single optical sections. Images were taken at a distance of 200 μm from the optic disc and within fields with a size of 300 × 800 μm^2^, which was modified from previous studies that chose the area of approximately 200–500 μm away from the optic nerve for quantification (Berger et al., 2014; Mead et al., 2014). Five images per retina were analyzed in three mice per group.

### Statistical Analysis

All experimental data were assessed by an operator blinded to the genetic condition. The results were presented as the mean ± standard error of the mean (SEM) and statistical significance was determined by independent Student’s *t*-test. *P* < 0.05 was considered significant. All analyses were performed using SPSS (IBM SPSS Statistics for Windows, Version 21.0, IBM Corp. Armonk, NY, United States) and GraphPad Prism 5.0a (GraphPad Software Inc., San Diego, CA, United States).

## Results

### Generation of Conditional Knockout Mice

To detect the *C-Ret^*lx/lx*^* allele in both *Chx10-Cre*;*C-Ret^*lx/lx*^* and *C-Ret^*lx/lx*^* mice, total DNA from wild-type (WT) and transgenic mice was subjected to PCR analysis with *C-Ret* primers spanning the intron 12 loxP sequence (Figure 1A, top). In WT mice (without the loxP insertion), the 350 bp amplicon was detected using the C-*Ret* primers. In contrast, given that *C-Ret^*lx/lx*^* and *Chx10-Cre;C-Ret^*lx/lx*^* mice contained loxP sequences, they yielded an amplicon of 450 bp instead (Figure 1B, top). Because the *Chx10-Cre* mouse model (JAX: Stock No. 005105) was generated by Chx10 BAC transgenes using a “GFP/Cre translational fusion protein” (Figure 1A, middle), we used GFP primers to determine the presence of Cre. Consequently, we found that a 320 bp fragment was detected exclusively in *Chx10-Cre;C-Ret^*lx/lx*^* mice, and not in WT or *C-Ret^*lx/lx*^* mice (Figure 1B, bottom).

### Conditional Knockout *C-Ret* in Retina and Expression of *Chx10-Cre*

**FIGURE 2:**
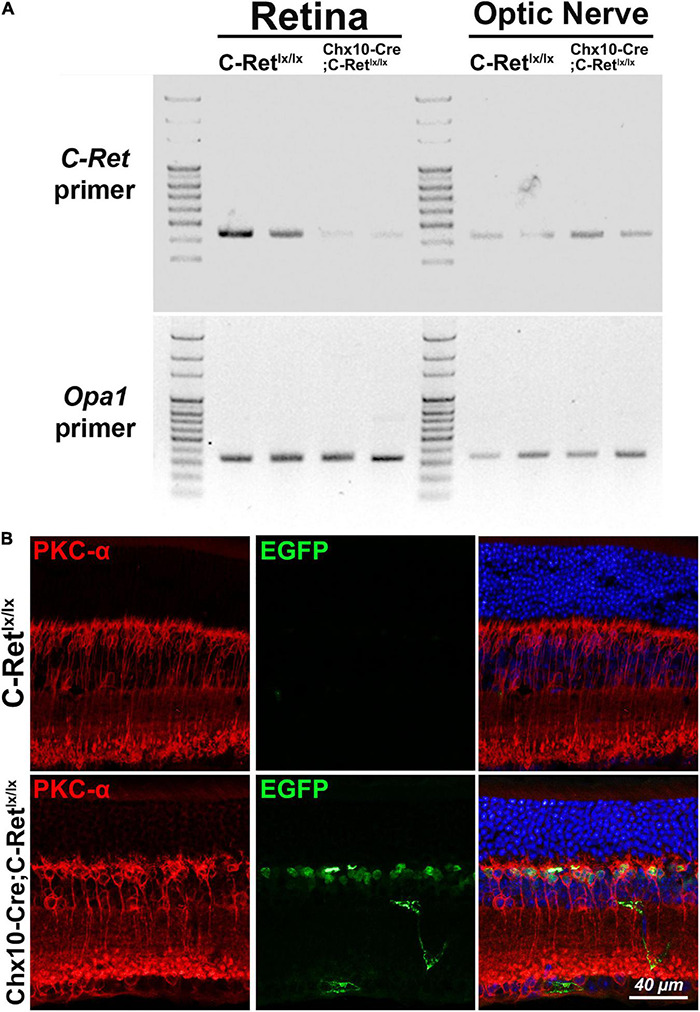
Amplification of *C-Ret* gene and immunohistochemistry of GFP in *C-Ret^*lx/lx*^* and *Chx10-Cre;C-Ret^*lx/lx*^* mice. (**A**, top) Polymerase chain reaction (PCR) was performed using *C-Ret* primers to screen the presence of exon 12 within the *C-Ret* gene. The results showed that barely any amplification occurred in the retinae of *Chx10-Cre;C-Ret^*lx/lx*^* mice compared to that in the retinae of *C-Ret^*lx/lx*^* mice. PCR was able to amplify *C-Ret* gene from optic nerve in both *C-Ret^*lx/lx*^* and *Chx10-Cre;C-Ret^*lx/lx*^* mice. (**A**, bottom) PCR analysis using *Opa1* primers showed equally amplification between *C-Ret^*lx/lx*^* and *Chx10-Cre;C-Ret^*lx/lx*^* mice in retinae and optic nerve tissue. **(B)** Retinal sections of animals aged 12 months were immunostained with anti-PKC-α (red) and anti-EGFP (green) antibodies to label rod bipolar and GFP expressing cells, respectively, followed by counterstaining with Hoechst dye, to indicate cell nuclei (blue). PKC-α (red) was expressed in cytoplasm of bipolar cells in *C-Ret^*lx/lx*^* and *Chx10-Cre;C-Ret^*lx/lx*^* mice; however, the GFP was only expressed in the nucleus of bipolar cells of *Chx10-Cre;C-Ret^*lx/lx*^* mice due to fusion with Cre protein, which has the capacity to cross the membrane and translocate to the nucleus.

To validate whether exon 12 of the *C-Ret gene* was deleted in the retina of *Chx10-Cre;C-Ret^*lx/lx*^* mice, we performed PCR analysis using *C-Ret* primers on DNA extracted from retina and optic nerve of *C-Ret^*lx/lx*^* and *Chx10-Cre;C-Ret^*lx/lx*^* mice. We found that PCR could amplify *C-Ret* in *C-Ret^*lx/lx*^* retina, but barely in *Chx10-Cre;C-Ret^*lx/lx*^* retina, indicating that exon 12 of the *C-Ret* gene was deleted in most of the retina cells of *Chx10-Cre;C-Ret^*lx/lx*^* mice (Figure 2A, top). On the other hand, PCR could amplify *C-Ret* from optic nerves in both *C-Ret^*lx/lx*^* and *Chx10-Cre;C-Ret^*lx/lx*^* mice. In addition, PCR amplification with *Opa1* primers was used to make sure the DNA was successfully extracted from different regions of eyeball and equally loading DNA amount for each lane (Figure 2A, bottom). To further validate the expression of Cre expression in *Chx10-Cre* mice, we did immunohistochemistry to label rod bipolar cells and GFP expressing cells using anti-PKC-α and anti-EGFP antibodies. Because the Cre protein on its own has the capacity to cross the membrane and translocate to the nucleus (Will et al., 2002), the IHC results showed GFP expression only in the nuclei of *Chx10-Cre;C-Ret^*lx/lx*^* bipolar cells, while PKC-α expression was identified in the cytoplasm of bipolar cells in both *C-Ret^*lx/lx*^* and *Chx10-Cre;C-Ret^*lx/lx*^* mice (Figure 2B). Based on PCR and IHC analysis, *Chx10-Cre;C-Ret^*lx/lx*^* mice were conditionally deleted the exon 12 of *C-Ret gene* in the retina.

### *In vivo* Analyses of Retinal Function and Morphology in *Chx10-Cre;C-Ret^*lx/lx*^* Mice

**FIGURE 3:**
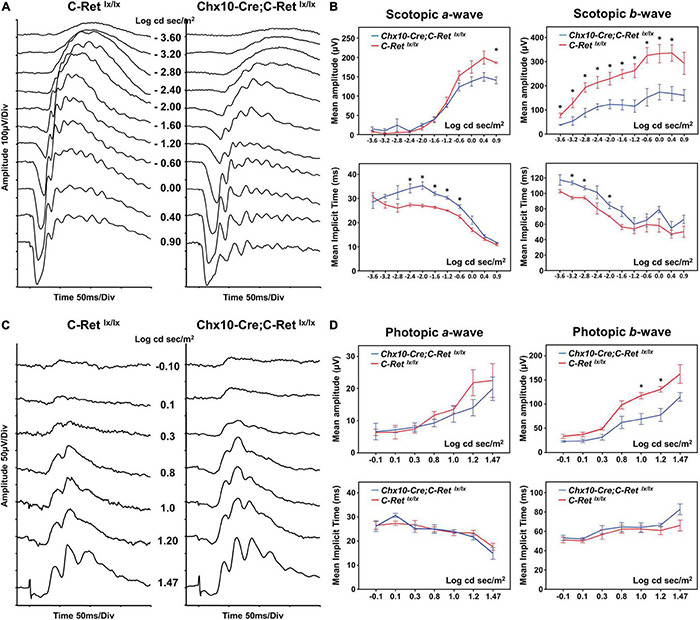
Electroretinography (ERG) of *C-Ret^*lx*/lx^* and *Chx10-CRE;C-Ret^*lx*/lx^* mice. **(A)** Representative ERG waveforms were recorded from 12-month-old *C-Ret^*lx/lx*^* and *Chx10-Cre;C-Ret^*lx/lx*^* mice at increasing stimulus intensities in the scotopic phase. In *Chx10-Cre;C-Ret^*lx/lx*^* mice, a decrease in the b-wave and a relative preservation of the a-wave were observed. **(B)** The b-wave amplitudes were significantly larger in *C-Ret^*lx/lx*^* mice than they were in *Chx10-Cre;C-Ret^*lx/lx*^* mice at most of the different intensities of the scotopic phase, whereas the a-wave amplitudes were similar between *C-Ret^*lx/lx*^* and *Chx10-Cre*;*C-Ret^*lx/lx*^* mice. There were some delays in the implicit time of a- and b- waves in *Chx10-Cre;C-Ret^*lx/lx*^* mice as compared to the implicit times in *C-Ret^*lx/lx*^* mice at some flash intensities. **(C)** Representative photopic ERG waveforms were recorded from 12-month-old *C-Ret^*lx/lx*^* and *Chx10-Cre;C-Ret^*lx/lx*^* mice at increasing stimulus intensities. **(D)** In *Chx10-Cre;C-Ret^*lx/lx*^* mice, there were reductions in b-wave amplitudes, while no significant decrease in the amplitude occurred in the a-wave. There were no significant delays in the a- and b- waves in photopic serial intensities. The electronegative ERG result indicated inner retinal dysfunction in *Chx10-Cre*;*C-Ret^*lx/lx*^* mice (*n* = 6; *P < 0.05).

To determine the effect of *Ret* deficiency on retinal function, ERG was performed in 12-month-old *Chx10-Cre;C-Ret^*lx/lx*^* and *C-Ret^*lx/lx*^* mice (Figure 3). The *C-Ret^*lx/lx*^* mice showed a normal ERG pattern of series intensity stimulation. As the flash intensity of the scotopic phase increased, the amplitude of the a-wave and b-wave increased. The a-wave represents the activity of the photoreceptors, whereas the b-wave reflects bipolar cell activity. The scotopic ERG of *Chx10-Cre;C-Ret^*lx/lx*^* mice revealed a selective reduction of b-waves, with relative preservation of a-waves in the scotopic phase (Figures 3A,B). The implicit times of a- and b- waves were more delayed in *Chx10-Cre;C-Ret^*lx/lx*^* mice (Figure 3B) (*n* = 6; ^∗^*P* < 0.05). In photopic serial intensity ERG, there were reductions in amplitude of a- and b- waves in *Chx10-Cre;C-Ret^*lx/lx*^* mice, which were statistically significant in some intensities of b-waves. However, there was no obvious difference in the implicit time of a- and b- waves in photopic responses between *Chx10-Cre;C-Ret^*lx/lx*^* and *C-Ret^*lx/lx*^* mice. The ERG recordings suggested that the *Chx10-Cre;C-Ret^*lx/lx*^* mice may exhibit a greater effect on the function of bipolar cells. Furthermore, these tracings were similar to those of an electronegative ERG corresponding to inner retinal dysfunction.

**FIGURE 4:**
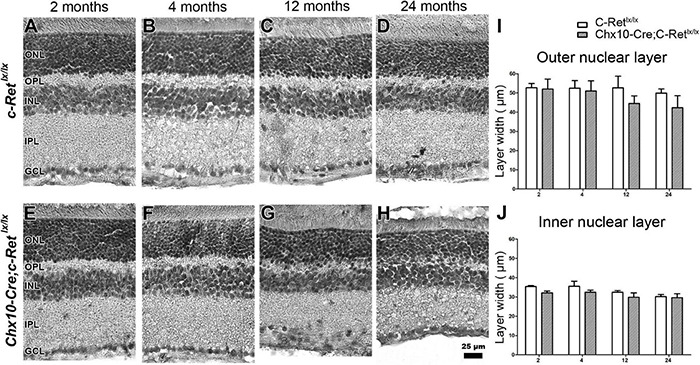
Morphological examination of *C-Ret^*lx*/lx^* and *Chx10-Cre;C-Ret^*lx/lx*^* mice. Hematoxylin staining of the retinas of *C-Ret^*lx*/lx^* **(A–D)** and *Chx10-Cre;C-Ret^*lx/lx*^* **(E–H)** mice was performed at 2, 4, 12, and 24 months of age. At the age of 2 and 4 months, the OPL in *Chx10-Cre;C-Ret^*lx/lx*^* retinas was slightly thinner than that in *C-Ret^*lx/lx*^* mice **(A,B,E,F)**. Furthermore, this phenomenon was detected in the OPL of *Chx10-Cre;C-Ret^*lx/lx*^* retinas which became worse, and their retina even showed disorganization at the ages of 12 and 24 months **(C,D,G,H)**. The thickness of the ONL and INL was measured in three animals per group at the ages of 2, 4, 12, and 24 months **(I,J)**. The ONL in *Chx10-Cre;C-Ret^*lx/lx*^* retinas was thinner than that in *C-Ret^*lx/lx*^* retinas at the ages of 12 and 24 months, albeit with no statistical significance at either of the stages **(I)**. Similarly, the INL in *Chx10-Cre;C-Ret^*lx/lx*^* retinas was slightly thinner (albeit not significantly) than that in *C-Ret^*lx/lx*^* retinas **(J)**. An independent *t*-test was used in this analysis, and significance was set at *P* < 0.05. ONL, outer nuclear layer; OPL, outer plexiform layer; INL, inner nuclear layer; IPL, inner plexiform layer; GCL, ganglion cell layer. Scale bar, 25 μm.

Hematoxylin staining was used to examine whether the *Chx10-Cre;C-Ret^*lx/lx*^* mice had morphological alterations in the retina (Figure 4). We observed that, compared with *C-Ret^*lx/lx*^* mice (Figures 4A–D), the retinas of *Chx10-Cre;C-Ret^*lx/lx*^* mice seemed to exhibit a progressive decrease in the thickness of the ONL, OPL, INL, and inner plexiform layer (IPL) (Figures 4E–H). Some nuclei in the ONL invaded the OPL in *Chx10-Cre;C-Ret^*lx/lx*^* mice at 12 months of age, which became more obvious in these animals at the age of 24 months. We then quantified the thickness of the ONL (Figure 4I) and INL (Figure 4J) using morphometric measurements. Although there was no statistically significant difference in the thickness of the ONL and INL between the *Chx10-Cre;C-Ret^*lx/lx*^* and *C-Ret^*lx/lx*^* retinas, we found that the thickness of the ONL in *Chx10-Cre;C-Ret^*lx/lx*^* mice showed a 15.4% reduction at 12 months and a 16.3% reduction at 24 months. This observation suggests that the retinal morphology in *Chx10-Cre;C-Ret^*lx/lx*^* mice is altered at older ages.

### Immunohistochemistry of Horizontal Cells and Rod Bipolar Cells in *Chx10-Cre;C-Ret^*lx/lx*^* Mice

**FIGURE 5:**
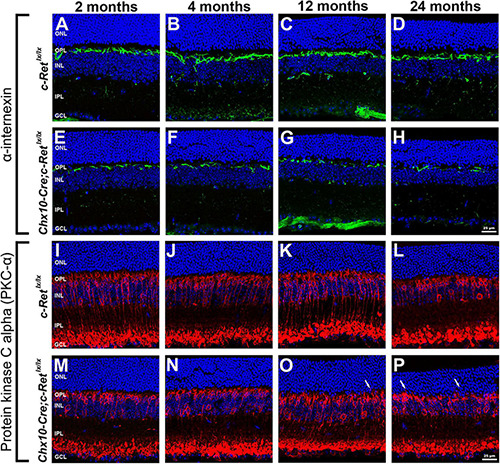
Immunohistochemistry of horizontal cells and rod bipolar cells in *C-Ret^*lx/lx*^* and *Chx10-Cre;C-Ret^*lx/lx*^* retinas. Retinal sections of mice aged 2, 4, 12, and 24 months were immunostained with an anti-α-internexin antibody (green, **A–H**) or an anti-PKC-α antibody (green, **I–P**), to label horizontal cells and rod bipolar cells, respectively, followed by counterstaining with Hoechst dye, to reveal cell nuclei (blue). Compared with *C-Ret^*lx/lx*^* mice **(A–D)**, the processes of horizontal cells were sparse and discontinuous in *Chx10-Cre;C-Ret^*lx/lx*^* mice and were even more deteriorated at the ages of 12 and 24 months **(E–H)**. The processes of rod bipolar cells were similar between *C-Ret^*lx/lx*^* **(I–L)** and *Chx10-Cre;C-Ret^*lx/lx*^* **(M–P)** mice aged 2, 4, 12, and 24 months. However, some processes of rod bipolar cells extended into the ONL at the ages of 12 and 24 months in *Chx10-Cre;C-Ret^*lx/lx*^* mice (**O,P**, arrows), which was not observed in *C-Ret^*lx/lx*^* mice. ONL, outer nuclear layer; OPL, outer plexiform layer; INL, inner nuclear layer; IPL, inner plexiform layer; GCL, ganglion cell layer. Scale bar, 25 μm.

To identify the components that are potentially altered in the OPL, IHC was performed to label the horizontal cells and rod bipolar cells in mouse retinas. Bipolar cells and horizontal cells are the second−order neurons that form synapses with photoreceptor terminals in the OPL. A previous study indicated that α-internexin is expressed in horizontal cells and may be used as a marker of these cells in the study of the mouse retina (Chien and Liem, 1995). Therefore, an anti-α-internexin antibody was used to detect the processes of horizontal cells (Figures 5A–H). In *C-Ret^*lx/lx*^* mice, horizontal cells possessed arborizing processes in the OPL of mice aged 2–24 months (Figures 5A–D). In contrast, the processes of horizontal cells in *Chx10-Cre;C-Ret^*lx/lx*^* mice were reduced at the ages of 2 and 4 months (Figures 5E,F), followed by a dramatic decrease at the ages of 12 and 24 months (Figures 5G,H). In order to obtain a second confirmation of changes in horizontal cells, the anti-calbindin antibody was applied to the sections, revealing a similar immunoreactivity pattern to that of α-internexin (Supplementary Figure 1). These results demonstrated that horizontal cells were affected in the conditional *Ret*-knockout mice.

The retinal rod bipolar cells expressed protein kinase C alpha (PKC-α), the distribution of which, within cells, is reportedly activity-dependent in the rat model (Gabriel et al., 2001). Thus, vertical sections of retinas were immunostained for PKC-α (Figures 5I–P). The immunoreactivity of PKC-α in *C-Ret^*lx/lx*^* mice showed that the bipolar cells had their cell bodies and the cytosolic compartments in the INL, the dendritic processes in the OPL, and the axon terminals in the innermost sublamina of the IPL (Figures 5I–L). A subpopulation of bipolar cells with axons terminating close to the ganglion cell layer was also observed. In *Chx10-Cre;C-Ret^*lx/lx*^* mice, bipolar cells exhibited a normal organization pattern, as in *C-Ret^*lx/lx*^* mice, and had well-preserved processes at the ages of 2 and 4 months (Figures 5M,N). However, at the ages of 12 and 24 months, some bipolar cells exhibited abnormal processes that sprouted into the ONL (Figures 5O,P). Furthermore, the aberrant processes tended to become longer and more numerous in aged *Chx10-Cre;C-Ret^*lx/lx*^* mice. According to these observations, bipolar cells may also be altered in conditional *Ret*-knockout mice.

### Alteration of Pre- and Post-synaptic Structures in *Chx10-Cre;C-Ret^*lx/lx*^* Retinas

**FIGURE 6:**
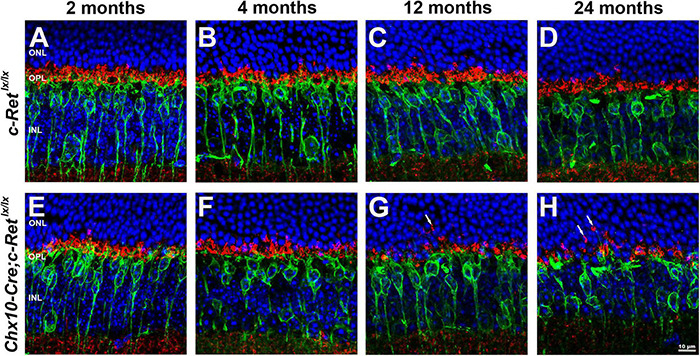
Immunohistochemistry of pre- and postsynaptic structures in *C-Ret^*lx*/lx^* and *Chx10-Cre;C-Ret^*lx/lx*^* retinas. Retinal sections of animals aged 2, 4, 12, and 24 months were immunostained with an anti-PKC-α antibody (green) and an anti-synaptophysin antibody (red), to label rod bipolar cells and the synapses of photoreceptors, respectively, followed by counterstaining with Hoechst dye, to indicate cell nuclei (blue) **(A–H)**. The processes of rod bipolar cells formed synapses with photoreceptors in the OPL of *C-Ret^*lx/lx*^* mice at all ages **(A–D)** and *Chx10-Cre;C-Ret^*lx/lx*^* mice at the ages of 2 and 4 months **(E–F)**. Ectopic synapses in the ONL were observed in 12- and 24-month-old *Chx10-Cre;C-Ret^*lx/lx*^* mice (**G,H**, arrows). ONL, outer nuclear layer; OPL, outer plexiform layer; INL, inner nuclear layer. Scale bar, 10 μm.

To determine whether the dendritic extensions of the rod bipolar cells change in synapses with photoreceptors, double IHC was performed. Given that synaptophysin is an integral membrane protein of synaptic vesicles, it is used to label synaptic structures in the terminals of photoreceptors (Nag and Wadhwa, 2001). Double immunohistochemistry using anti-PKC-α and anti-synaptophysin antibodies demonstrated that PKC-α-positive processes lay among synaptophysin-positive terminals in the OPL of *C-Ret^*lx/lx*^* mice (Figures 6A–D). A similar pattern was observed in *Chx10-Cre;C-Ret^*lx/lx*^* mice aged 2 and 4 months that processes, i.e., most of the rod bipolar cells labeled by the anti-PKC-α antibody were associated with synaptophysin-labeled rod axon terminals in the OPL (Figures 6E,F). However, in *Chx10-Cre;C-Ret^*lx/lx*^* mice aged 12 and 24 months, some PKC-α- and synaptophysin-positive processes were mislocalized in the ONL and synaptophysin-positive processes were also decreased (Figures 6G,H). In addition, the post-synaptic density protein 95 (PSD-95) was detected in the OPL (Supplementary Figure 2). A significant decrease of PSD-95 expression was found in *Chx10-Cre;C-Ret^*lx/lx*^* mice aged 12 and 24 months, compared to *C-Ret^*lx/lx*^* mice. The IHC results indicated that the synapses between rod bipolar cells and photoreceptors were affected in conditional *Ret*-knockout mice.

**FIGURE 7:**
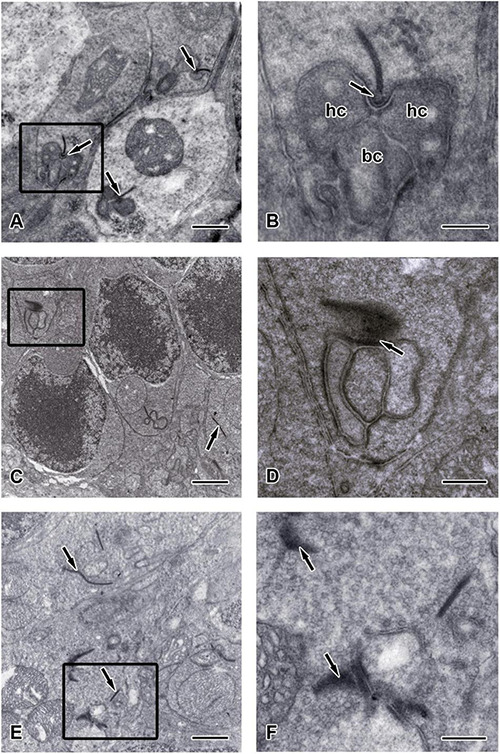
Ultrastructure of the synaptic ribbons in *C-Ret^*lx/lx*^* and *Chx10-Cre;C-Ret^*lx/lx*^* retinas at the age of 2 months. Transmission electron microscopy (TEM) images showed the outer plexiform layer of *C-Ret^*lx/lx*^* **(A,B)** and *Chx10-Cre;C-Ret^*lx/lx*^* **(C–F)** mice at the age of 2 months. **(A)** Three typical rod-like synaptic ribbon profiles (arrows) with several mitochondria were observed in the pedicle of *C-Ret^*lx/lx*^* mice. **(B)** Higher magnification of the TEM image is shown in the rectangle of **(A)**. The electron dense synaptic ribbon was surrounded by a halo of synaptic vesicles. The arciform density (arrows) was positioned between the base of the ribbon and the plasma membrane. Three post-synaptic processes, two dendritic tips of horizontal cells (hc) and one dendritic tips of bipolar cells (bc), were closely apposed to the photoreceptor near the ribbon. **(C,E)** Most synaptic ribbons (arrows) in the rod spherules appeared to float in the cytoplasm, unassociated with an arciform density and the presynaptic membrane. **(D)** Higher magnification of the TEM image is shown in the rectangle of **(C)**. Synaptic structure in pedicle (arrow) of *Chx10-Cre;C-Ret^*lx/lx*^* retina was found to be dramatically different from that in the *C-Ret^*lx/lx*^* retina. **(F)** Higher magnification of the TEM image is shown in the rectangle of **(E)**. “Medusa-like” ribbons (arrows) were observed in which they displayed fewer postsynaptic processes that appeared to invaginate into a photoreceptor terminal. Scale bars: **(A,C,E)**, 1 μm; **(B,D,F)**, 300 nm.

**FIGURE 8:**
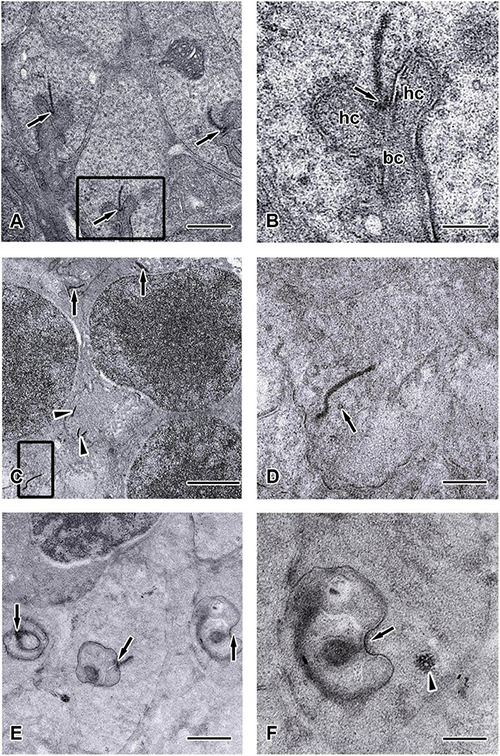
Ultrastructure of the synaptic ribbons in *C-Ret^*lx/lx*^* and *Chx10-Cre;C-Ret^*lx/lx*^* retinas at the age of 12 months. TEM images showed the outer plexiform layer (ONL) of *C-Ret^*lx/lx*^* **(A,B)** and *Chx10-Cre;C-Ret^*lx/lx*^* **(C–F)** mice at the age of 12 months. **(A)** Three typical rod-like synaptic ribbon profiles (arrows) with several mitochondria were observed in the rod spherules of *C-Ret^*lx/lx*^* mice. **(B)** Higher magnification of the TEM image is shown in the rectangle of **(A)**. The large presynaptic terminals were filled with numerous synaptic vesicles, and the active zone was characterized by specialized presynaptic densities, the arciform densities (arrows). Opposite to the active zones were the dendritic tips of horizontal cells (hc) and bipolar cells (bc), which contained ionotropic and metabotropic glutamate receptors for signaling. **(C–E)** Electron micrographs showed different examples of rod terminals and the ultrastructural appearance of the ribbon synaptic complex in the *Chx10-Cre;C-Ret^*lx/lx*^* retina. **(C)** Some synaptic ribbons (arrows) were found to be mislocalized between cell nuclei in ONL, which were supposed to be found in outer plexiform layer. All other synaptic ribbons were found floating in the cytoplasm (arrowheads) and did not associate with an arciform density nor with the presynaptic membrane. **(D)** Higher magnification of the TEM images is shown in the rectangle of **(C)**. Orphan presynaptic density surrounded by synaptic vesicles (arrow) and irregularly shaped floating ribbons were shown in *Chx10-Cre;C-Ret^*lx/lx*^* retinae. **(E)** Three ultrastructural appearance of the ribbon synaptic complexes (arrows) of photoreceptor terminals were shown. **(F)** Higher magnification of the TEM images, shown in the rectangles of **(E)**, displayed an abnormal terminal with postsynaptic elements but without presynaptic ribbon (arrow). Although synaptic vesicles could still be observed, they seemed to be aggregated (arrowhead) rather than in their typical, individual form. Scale bars: **(A,E)**, 1 μm; **(B,D,F)**, 300 nm; **(C)**, 2 μm.

To further confirm the morphological changes observed in the OPL of *Chx10-Cre*;*C-Ret^*lx/lx*^* mice, we performed TEM observations and identified ribbon synapses at the outer retina of *C-Ret^*lx/lx*^* and *Chx10-Cre;C-Ret^*lx/lx*^* mice aged 2 months (Figure 7) and 12 months (Figure 8). The photoreceptor ribbon synapses of *C-Ret^*lx/lx*^* mice exhibited a varying number of rod-shaped profiles in photoreceptor terminals (Figures 7A,B). Ribbon ultrastructure was defined by the central presynaptic ribbon opposed by two postsynaptic horizontal cell processes. The synaptic ribbon displayed its typical plate-like shape, extending perpendicular to the presynaptic membrane into the cytoplasm. Although, plentiful synaptic ribbons in *Chx10-Cre;C-Ret^*lx/lx*^* mice aged 2 months, having roughly the same length and appearance as those in *C-Ret^*lx/lx*^* terminals, were observed, they were usually found to be “floating” in the cytoplasm instead of anchored to other synaptic structures (Figures 7C–E). Besides, the number of “Medusa-like’ ribbons seemed to be increased and the synaptic ribbons were observed reduced in height and swollen shaped (Figures 7E,F). Therefore, the synaptic ribbon is surrounded by a halo of synaptic vesicles, as they do in the *C-Ret^*lx/lx*^* retina.

**FIGURE 9:**
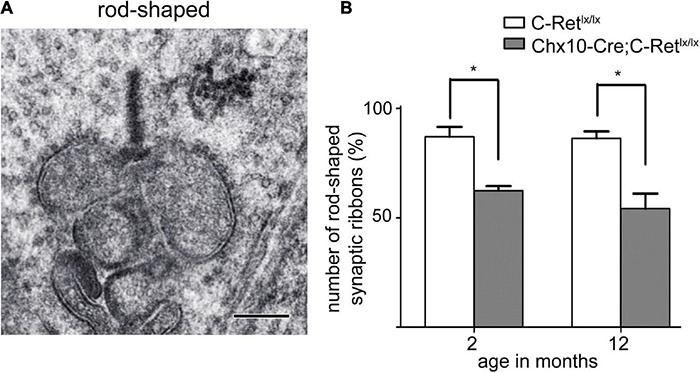
Rod-shaped synaptic ribbons of photoreceptors disintegrated in *Chx10-Cre;C-Ret^*lx/lx*^* mice. **(A)** A typical rod-shaped synaptic ribbon is shown in the large presynaptic terminal. Scale bar: 300 nm. **(B)** Quantification was performed to investigate the number of rod-shaped synaptic ribbons in retinae of *C-Ret^*lx/lx*^* and *Chx10-Cre;C-Ret^*lx/lx*^* mice at the age of 2 and 12 months. The histogram was plotted using mean values ± standard error of the mean (SEM). * Denotes *p* < 0.05 in Student’s *t*-test (*n* = 3).

Furthermore, the appearance of the ribbon synaptic complexes in the 12-month *Chx10-Cre*;*C-Ret^*lx/lx*^* mice retinae differed significantly from those in the *C-Ret^*lx/lx*^* retinae. Normally, a rod synaptic terminal in *C-Ret^*lx/lx*^* retina contained a single ribbon synaptic site (Figure 8A), where glutamate was released onto the postsynaptic elements, horizontal cell processes and rod bipolar cell dendrites. The postsynaptic elements invaginated into the rod terminal and formed a triadic or tetradic configuration adjacent to the ribbon site (Figure 8B). However, most of the ribbons in the retinae of 12-month-old *Chx10-Cre*;*C-Ret^*lx/lx*^* mice were not docked at the synaptic site (i.e., they floated freely in the cytoplasm) (Figures 8C,D). Many empty rod terminals without presynaptic ribbons and postsynaptic invaginating elements were found in *Chx10-Cre*;*C-Ret^*lx/lx*^* mice (Figure 8E). In addition, the number of synaptic vesicles was decreased near synapses (Figure 8D) and found in clumps rather than distributed evenly in the pedicle (Figure 8F). Following these observations, we classified the synaptic ribbons into two categories: rod-shaped and non-rod-shaped, based on their general morphological features; this was followed by quantification of the synaptic ribbons. Representative examples of the rod-shaped ribbons anchored at the active zone, where exocytosis of synaptic vesicles occurred, are shown in Figure 9A and the quantitative data are summarized in Figure 9B. 87.1 and 86.3% of rod photoreceptor ribbon profiles were presynaptically anchored and rod-shaped in *C-Ret^*lx/lx*^* mice aged 2 and 12 months, respectively. In contrast, a significant decrease was found in the *Chx10-Cre;C-Ret^*lx/lx*^* mice whereby only 62.4% of rod photoreceptor ribbon profiles were presynaptically anchored and rod-shaped at the age of 2 months; this proportion only worsened at the age of 12 months (54.2% of rod photoreceptor ribbons). The aforementioned TEM results suggest that the loss of *Ret* causes a structural defect in the synaptic connection between photoreceptors and bipolar cells. Ultimately, this finding may underlie the abnormal ERG b-wave observed in *Chx10-Cre;C-Ret^*lx/lx*^* mice.

## Discussion

In this study, we generated retinal *Ret*-specific knockout mice using the Cre/loxP system and demonstrated a possible role for *Ret* in retinal function. First, we found severely abnormal ERG patterns, especially those of b-waves, in conditional *Ret*-knockout retinas. Second, we identified gradually reduced levels of immunoreactivity for α-internexin—a marker of processes of horizontal cells—as *Chx10-Cre;C-Ret^*lx/lx*^* mice increased in age. Third, deficiency of *Ret* in the retina caused the mislocalization of synapses in the ONL, as demonstrated by immunostaining for PKC-α and synaptophysin. Finally, ultrastructural observations of conditional *Ret*-knockout retinas revealed that the synaptic ribbons were immature and not fully assembled, which may explain the abnormal ERG results.

The function of *Ret* in the retina was well assessed by full-field ERG in previous studies. *Ret* hypomorphic mice, which exhibit severely reduced *Ret* activity, do not survive beyond 3 weeks and display significantly reduced scotopic a-waves, b-waves, and photopic b-waves at postnatal day 18 (Brantley et al., 2008). In this study, the *Chx10-Cre;C-Ret^*lx/lx*^* mice, which had conditional retinal *Ret* deficiency, exhibited a prolonged survival time and a selective reduction in b-waves, but normal a-waves, on ERG performed at 12 months of age. As such, this waveform was deemed an electronegative ERG and suggested a dysfunction of the ON bipolar cells. In addition to bipolar cells, it was also hypothesized that reduced b-waves could result from the impaired horizontal cells if their inhibition to bipolar cells was switched off (Goetze et al., 2010). Taken together, these results indicate that sufficient *Ret* expression is required for normal retinal function and development.

GDNF and other GFLs (ARTN, NRTN, and PSPN) share the RET receptor tyrosine kinase as their common signaling receptor. A previous study revealed a thinner and disorganized OPL in *NRTN^–/–^* mice, suggesting that the aberrant morphology of photoreceptors, bipolar cells, and horizontal cells was caused by NRTN deficiency (Brantley et al., 2008). Moreover, GDNF can increase the proliferation, promote the differentiation, and prevent the programmed death of chicken rod photoreceptors, as assessed using re-aggregated retinal spheroids as an *in vitro* assay model (Rothermel and Layer, 2003). Another GDNF-family receptor alpha-4 (GFRα4)-deficient retinal culture study showed a decrease in the number of amacrine cells, horizontal cells, and blue-sensitive cone photoreceptors in this system (Rothermel et al., 2006). In our study, the immunoreactivities of α-internexin and PKC-α were altered in *Chx10-Cre;C-Ret^*lx/lx*^* mice. These results indicate that *Ret* dysfunction may trigger an abnormal morphology in horizontal cells and bipolar cell processes, and provided evidence that GFLs, GDNF-family receptors (GFRs), or the RET receptor tyrosine kinase can specifically affect distinct photoreceptors and other retinal cell subpopulations.

It was reported that GDNF can be produced by glial cells to increase the survival rate of a retinal ganglion or photoreceptor cells in different experimental models, such as the rescue of retinal ganglion cells after axotomy (Koeberle and Ball, 1998), the delivery of a protective effect in mice with glaucoma (Johnson et al., 2011), or the protection of photoreceptors in the *rd1* mouse (Frasson et al., 1999). Furthermore, GDNF also moderately protected the rat retina from ischemia–reperfusion injury, possibly by preventing apoptosis in retinal cells (Wu et al., 2004). These previous studies suggested that, in the absence of GDNF, retinal cells lose a protective factor, which might lead to serious retinal dysfunction or degeneration. In addition, a previous study demonstrated that GDNF partially restored ureteric branching morphogenesis in *Ret*-deficient mice with severe renal hypodysplasia, possibly through the induction of Met phosphorylation, rather than through RET signaling (Popsueva et al., 2003). This implies that it is also possible that GDNF partially signals independently of RET through the GDNF-family receptor alpha-1 (GFRα1) and Met phosphorylation in the retina. In fact, the GFL–GFRα1 complex activates Met kinase indirectly via Src kinases in the absence of RET kinase (Popsueva et al., 2003). The GFLs can also interact directly with heparan sulfate proteoglycans to activate Met kinase, which might be mediated by a neural cell adhesion molecule (N-CAM) (Sariola and Saarma, 2003). Our results revealed that the thickness of the ONL was decreased at the stage of 12 and 24 months; however, there were no significant differences between *C-Ret^*lx/lx*^* and *Chx10-Cre;C-Ret^*lx/lx*^* mice. Moreover, the processes of horizontal cells were significantly reduced, and the immunoreactivities of synaptophysin and PKC-α demonstrated the presence of mislocalized synapses in the ONL. Despite these alterations, the retinas of *Chx10-Cre;C-Ret^*lx/lx*^* mice did not show severe disorganization. We speculated that GDNF signaling independently of RET and via GFRα1 might explain why the retinas of *Chx10-Cre;C-Ret^*lx/lx*^* mice failed to show severe dysfunction or disorganization.

GDNF is also known as one of the neurotrophic factors that play key roles in the development and survival of neurons. Neurotrophic factors generally include the neurotrophin family [NGF, BDNF, neurotrophin-3 (NT-3), and NT-4/5], the GDNF family (GDNF, NRTN, ARTN, and PSPN), and the CNTF, which is a member of the interleukin 6 (IL-6) family of cytokines. Different factors can act in a sequential, simultaneous, additive (synergistical), or mutual-inhibition fashion. For instance, subpopulations of developing sensory and motor neurons are dependent on the simultaneous action of GDNF and BDNF (Henderson, 1996). Moreover, a combination of GDNF and CNTF was reported to afford higher protection to photoreceptors in a retinal degeneration (rd) mouse (Ogilvie et al., 2000). In our study, *Chx10-Cre;C-Ret^*lx/lx*^* mouse retinas had deletion of RET, which is the canonical GDNF receptor, but did not show severe dysfunction or disorganization. According to the studies mentioned above, another explanation for the resulting mild dysfunction following RET deletion is that the remaining neurotrophic factors—which remained unaffected—compensated for the effects of knocking out the RET signaling pathway. However, this study did not investigate the neurotrophic factors or possible signaling pathways independent of RET that may play roles in the retinas of *Chx10-Cre;C-Ret^*lx/lx*^* mice. Therefore, further research is required to clarify the mechanisms underlying these observations.

The ribbon complex of retinal photoreceptor synapses represents a specialization of the cytomatrix at the active zone that is present at conventional synapses. The active zones of synapses are highly organized structures designed for the regulated and site-specific release of neurotransmitters. The function of photoreceptor ribbons was suggested to be the continuous shuttling of vesicles to the active zone, for fusion and the release of glutamate (Lenzi and von Gersdorff, 2001). In a previous study, ribbons in *Bassoon (Bsn)*-mutant mouse retinas did not attach to the active zone, thus potentially resulting in the failure of synaptic transmission. Moreover, the a-waves of the ERG recordings performed in the *Bsn*-mutant mouse were not affected, whereas their b-waves, representing the response of the ON bipolar cells, were significantly reduced in amplitude and prolonged in implicit time (Dick et al., 2003). Our ultrastructural observation revealed the inappropriate assembly of synaptic ribbons, which then failed to anchor themselves to the active zone. Moreover, a severely affected ERG was recorded in the retinas of *Chx10-Cre;C-Ret^*lx/lx*^* mice, implying that conditional deletion of *Ret* in the retina may cause a dysfunction in synaptic transmission. In addition, other findings demonstrated that the co-administration of the fibroblast growth factor 2 (FGF-2) and GDNF can promote the long-term survival of target-deprived adult mouse spiral ganglion neurons (Wei et al., 2007). Furthermore, GDNF was shown to contribute to synaptic development and maturation in ventral midbrain dopaminergic neurons and spinal cord motoneurons (Bourque and Trudeau, 2000). Therefore, conditional deletion of *Ret*, the canonical GDNF receptor, may affect retinal development and cause morphological and physiological alterations in *Chx10-Cre;C-Ret^*lx/lx*^* mouse retinas.

Our study has documented dendritic sprouting in aging Ret-deficient mouse retinae. The outgrowth of bipolar cell dendrites was reported to be observed under some pathologic conditions, such as retinal detachment (Fisher et al., 2005), the nob2 mouse with a calcium channel Cav1.4 null mutation (Bayley and Morgans, 2007) and Bsn mice lacking functional Bassoon protein (Dick et al., 2003). A study of the RCS rat whose retina underwent progressive photoreceptor degeneration also demonstrated dendritic sprouting of rod bipolar cells (Cuenca et al., 2005). Furthermore, NRTN-deficient mice—a model deficit in one of GDNF family ligands—also showed abnormally located synapses in the ONL (Brantley et al., 2008). This study also suggested that the abnormal synapse formation in the ONL caused deficits in the signaling of photoreceptor to bipolar cell and contributed to the ERG defects which was similar to what we have observed at 2–24 month of *Chx10-Cre;C-Ret^*lx/lx*^* mice. However, a previous study indicated that the dendrites of rod bipolar cells, normally confined to the OPL, were found to extend into the ONL in normal aging retina and tended to increase in length and incidence with the age (Liets et al., 2006). Although the effect of aging could not be excluded in the abnormal synaptic formation of the Ret-deficient retina, the results of our ERG recordings, comparing *C-Ret^*lx/lx*^* and *Chx10-Cre;C-Ret^*lx/lx*^* mice at the age of 12 months, indicated that the *Chx10-Cre;C-Ret^*lx/lx*^* mice may have dysfunctional bipolar cells. Additionally, a previous study speculated that reduced synaptic efficacy may induce new neuronal growth and the formation of ectopic synapses in *Basson* mutant mice (Dick et al., 2003). Therefore, combining our ERG and morphological results, we deduced that inadequate Ret expression may increase the formation of ectopic synapses and exacerbate these to a dysfunctional level. While not evaluated here, further studies should look to investigate the specific molecular mechanisms mediating the genesis and function of ectopic synapses in Ret-deficient mice.

## Conclusion

In conclusion, our results provide evidence of the role of *Ret* in retinal development, which is essential to maintain the processes of horizontal cells and preserve the integrity of the OPL by stabilizing the structure of the synaptic ribbons. The *Chx10-Cre;C-Ret^*lx/lx*^* mice developed in this study provided a valuable model in which to study *Ret* function in the retina and enhanced the understanding of *Ret* function in postnatal development and later stages. Finally, these conditional *Ret*-knockout mice might be useful for investigating the importance of GFL-mediated RET activation in the retina of animal models of other diseases, such as neurodegenerative diseases or genetic disorders.

## Data Availability Statement

The raw data supporting the conclusions of this article will be made available by the authors, without undue reservation.

## Ethics Statement

The animal study was reviewed and approved by the National Taiwan University Animal Ethics Committee.

## Author Contributions

W-HP conceived of the study, carried out the ultrastructure observation, drafted the manuscript, and final approval of the manuscript submission. M-LL participated in the design of the experiment and edited the manuscript. W-CH and C-YL carried out the antibody sensitivity test, immunohistochemical staining, and PCR experiment. SL edited the manuscript. Y-JT contributed with the electroretinography and data analysis. L-KY and K-JC contributed with the data analysis and interpretation. C-LC and N-KW designed the study, revised the work critically for important intellectual content, and gave their final approval of the version to be published. All the authors contributed to and approved the final manuscript.

## Conflict of Interest

The authors declare that the research was conducted in the absence of any commercial or financial relationships that could be construed as a potential conflict of interest.

## Publisher’s Note

All claims expressed in this article are solely those of the authors and do not necessarily represent those of their affiliated organizations, or those of the publisher, the editors and the reviewers. Any product that may be evaluated in this article, or claim that may be made by its manufacturer, is not guaranteed or endorsed by the publisher.

## Supplementary Material

Supplementary Figure 1 Calbindin staining of horizontal cells in *C-Ret^*lx/lx*^* and *Chx10-Cre;C-Ret^*lx/lx*^* retinas. Immunostaining against calbindin (green), a specific marker for horizontal cells, followed by counterstaining with Hoechst dye to indicate cell nuclei (blue) is shown. Compared to those of *C-Ret^*lx/lx*^* mice **(A–D)**, confocal microscopic images of horizontal cells at 2 month **(E)**, 4 months **(F)**, 12 months **(G)**, and 24 months **(H)** of *Chx10-Cre;C-Ret^*lx/lx*^* mice displayed a progressed decrease in calbindin-positive immunoreactivity with an increase in age. ONL, outer nuclear layer; OPL, outer plexiform layer; INL, inner nuclear layer; IPL, inner plexiform layer; GCL, ganglion cell layer. Scale bar, 25 μm.

Supplementary Figure 2 Mislocalization of photoreceptor synaptic terminals observed in *Chx10-Cre;C-Ret^*lx/lx*^* mice. Retinal sections of animals aged 2, 4, 12, and 24 months were immunostained with an anti-PKC-α antibody (green) and an anti-PSD95 antibody (red), to label rod bipolar cells and the synapses of photoreceptors, respectively, followed by counterstaining with Hoechst dye, to indicate cell nuclei (blue) **(A–H)**. The processes of rod bipolar cells formed synapses with photoreceptors in the OPL of *C-Ret^*lx/lx*^* mice at all ages **(A–D)** and *Chx10-Cre;C-Ret^*lx/lx*^* mice at the ages of 2 and 4 months **(E–F)**. However, extended rod bipolar cell dendrites and ectopic photoreceptor terminals (arrows) in the ONL were observed in 12- and 24-month-old *Chx10-Cre;C-Ret^*lx/lx*^* mice **(G,H)**. ONL, outer nuclear layer; OPL, outer plexiform layer; INL, inner nuclear layer. Scale bar, 10 μm.

## Abbreviations

ARTN: artemin
BDNF: brain-derived neurotrophic factor
CNTF: ciliary neurotrophic factor
ERG: electroretinography
GDNF: glial-cell-line-derived neurotrophic factor
GFLs: GDNF-family ligands
INL: inner nuclear layer
IPL: inner plexiform layer
NGF: nerve growth factor
NRTN: neurturin
ONL: outer nuclear layer
OPL: outer plexiform layer
PSPN: persephin
RT: room temperature
TGF- β: transforming growth factor β
WT: wild-type.

## References

[B1] Airaksinen M. S. Saarma M. (2002). The GDNF family: signalling, biological functions and therapeutic value. *Nat. Rev. Neurosci.* 3 383–394. 10.1038/nrn812 11988777

[B2] Bayley P. R. Morgans C. W. (2007). Rod bipolar cells and horizontal cells form displaced synaptic contacts with rods in the outer nuclear layer of the nob2 retina. *J. Comp. Neurol.* 500 286–298. 10.1002/cne.21188 17111373 PMC4238417

[B3] Berger A. Cavallero S. Dominguez E. Barbe P. Simonutti M. Sahel J.-A. (2014). Spectral-domain optical coherence tomography of the rodent eye: highlighting layers of the outer retina using signal averaging and comparison with histology. *PLoS One* 9:e96494. 10.1371/journal.pone.0096494 24788712 PMC4008571

[B4] Bourque M. J. Trudeau L. E. (2000). GDNF enhances the synaptic efficacy of dopaminergic neurons in culture. *Eur. J. Neurosci.* 12 3172–3180. 10.1046/j.1460-9568.2000.00219.x 10998101

[B5] Brantley M. A. Jr. Jain S. Barr E. E. Johnson E. M. Jr. Milbrandt J. (2008). Neurturin-mediated ret activation is required for retinal function. *J. Neurosci.* 28 4123–4135. 10.1523/JNEUROSCI.0249-08.2008 18417692 PMC2704905

[B6] Chien C. L. Liem R. K. (1995). The neuronal intermediate filament, alpha-internexin is transiently expressed in amacrine cells in the developing mouse retina. *Exp. Eye Res.* 61 749–756. 10.1016/s0014-4835(05)80026-0 8846847

[B7] Chinskey N. D. Besirli C. G. Zacks D. N. (2014). Retinal cell death and current strategies in retinal neuroprotection. *Curr. Opin. Ophthalmol.* 25 228–233. 10.1097/ICU.0000000000000043 24614145

[B8] Costantini F. Shakya R. (2006). GDNF/Ret signaling and the development of the kidney. *Bioessays* 28 117–127. 10.1002/bies.20357 16435290

[B9] Cuenca N. Pinilla I. Sauvé Y. Lund R. (2005). Early changes in synaptic connectivity following progressive photoreceptor degeneration in RCS rats. *Eur. Neurosci.* 22 1057–1072. 10.1111/j.1460-9568.2005.04300.x 16176347

[B10] Dick O. Tom Dieck S. Altrock W. D. Ammermüller J. Weiler R. Garner C. C. (2003). The presynaptic active zone protein bassoon is essential for photoreceptor ribbon synapse formation in the retina. *Neuron* 37 775–786. 10.1016/s0896-6273(03)00086-2 12628168

[B11] Durbec P. Marcos-Gutierrez C. V. Kilkenny C. Grigoriou M. Wartiowaara K. Suvanto P. (1996). GDNF signalling through the Ret receptor tyrosine kinase. *Nature* 381 789–793. 10.1038/381789a0 8657282

[B12] Ernsberger U. (2008). The role of GDNF family ligand signalling in the differentiation of sympathetic and dorsal root ganglion neurons. *Cell Tissue Res.* 333 353–371. 10.1007/s00441-008-0634-4 18629541 PMC2516536

[B13] Errijgers V. Van Dam D. Gantois I. Van Ginneken C. J. Grossman A. W. D’Hooge R. (2007). FVB.129P2-Pde6b(+) Tyr(c-ch)/Ant, a sighted variant of the FVB/N mouse strain suitable for behavioral analysis. *Genes Brain Behav.* 6 552–557. 10.1111/j.1601-183X.2006.00282.x 17083330

[B14] Fisher S. K. Lewis G. P. Linberg K. A. Verardo M. R. (2005). Cellular remodeling in mammalian retina: results from studies of experimental retinal detachment. *Prog. Retin. Eye Res.* 24 395–431. 10.1016/j.preteyeres.2004.10.004 15708835

[B15] Frasson M. Picaud S. Leìveillard T. Simonutti M. Mohand–Said S. Dreyfus H. (1999). Glial cell line–derived neurotrophic factor induces histologic and functional protection of rod photoreceptors in the rd/rd mouse. *Investig. Ophthalmol. Vis. Sci.* 40 2724–2734. 10509671

[B16] Gabriel R. Lesauter J. Silver R. Garcia-Espana A. Witkovsky P. (2001). Diurnal and circadian variation of protein kinase C immunoreactivity in the rat retina. *J. Comp. Neurol.* 439 140–150. 10.1002/cne.1338 11596044 PMC3271847

[B17] Goetze B. Schmidt K. F. Lehmann K. Altrock W. D. Gundelfinger E. D. Lowel S. (2010). Vision and visual cortical maps in mice with a photoreceptor synaptopathy: reduced but robust visual capabilities in the absence of synaptic ribbons. *Neuroimage* 49 1622–1631. 10.1016/j.neuroimage.2009.10.019 19837175

[B18] Heidelberger R. Thoreson W. B. Witkovsky P. (2005). Synaptic transmission at retinal ribbon synapses. *Prog. Retin. Eye Res.* 24 682–720. 10.1016/j.preteyeres.2005.04.002 16027025 PMC1383430

[B19] Henderson C. E. (1996). Role of neurotrophic factors in neuronal development. *Curr. Opin. Neurobiol.* 6 64–70. 10.1016/s0959-4388(96)80010-9 8794045

[B20] Henderson C. E. Phillips H. S. Pollock R. A. Davies A. M. Lemeulle C. Armanini M. (1994). GDNF: a potent survival factor for motoneurons present in peripheral nerve and muscle. *Science (New York, NY)* 266 1062–1064. 10.1126/science.7973664 7973664

[B21] Johnson T. V. Bull N. D. Martin K. R. (2011). Neurotrophic factor delivery as a protective treatment for glaucoma. *Exp. Eye Res.* 93 196–203. 10.1016/j.exer.2010.05.016 20685205

[B22] Koeberle P. D. Ball A. K. (1998). Effects of GDNF on retinal ganglion cell survival following axotomy. *Vision Res.* 38 1505–1515. 10.1016/s0042-6989(97)00364-7 9667015

[B23] Kramer E. R. Aron L. Ramakers G. M. Seitz S. Zhuang X. Beyer K. (2007). Absence of Ret signaling in mice causes progressive and late degeneration of the nigrostriatal system. *PLoS Biol.* 5:e39. 10.1371/journal.pbio.0050039 17298183 PMC1808500

[B24] Kramer E. R. Knott L. Su F. Dessaud E. Krull C. E. Helmbacher F. (2006). Cooperation between GDNF/Ret and ephrinA/EphA4 signals for motor-axon pathway selection in the limb. *Neuron* 50 35–47. 10.1016/j.neuron.2006.02.020 16600854

[B25] Lenzi D. von Gersdorff H. (2001). Structure suggests function: the case for synaptic ribbons as exocytotic nanomachines. *Bioessays* 23 831–840. 10.1002/bies.1118 11536295

[B26] Liets L. C. Eliasieh K. van der List D. A. Chalupa L. M. (2006). Dendrites of rod bipolar cells sprout in normal aging retina. *Proc. Natl. Acad. Sci. U.S.A.* 103:12156. 10.1073/pnas.0605211103 16880381 PMC1524926

[B27] Lin L. F. Doherty D. H. Lile J. D. Bektesh S. Collins F. (1993). GDNF: a glial cell line-derived neurotrophic factor for midbrain dopaminergic neurons. *Science (New York, NY)* 260 1130–1132. 10.1126/science.8493557 8493557

[B28] LoGiudice L. Matthews G. (2009). The role of ribbons at sensory synapses. *Neuroscientist* 15 380–391. 10.1177/1073858408331373 19264728 PMC2743156

[B29] Mattapallil M. J. Wawrousek E. F. Chan C. C. Zhao H. Roychoudhury J. Ferguson T. A. (2012). The Rd8 mutation of the Crb1 gene is present in vendor lines of C57BL/6N mice and embryonic stem cells, and confounds ocular induced mutant phenotypes. *Invest. Ophthalmol. Vis. Sci.* 53 2921–2927. 10.1167/iovs.12-9662 22447858 PMC3376073

[B30] Mead B. Thompson A. Scheven B. A. Logan A. Berry M. Leadbeater W. (2014). Comparative evaluation of methods for estimating retinal ganglion cell loss in retinal sections and wholemounts. *PLoS One* 9:e110612. 10.1371/journal.pone.0110612 25343338 PMC4208790

[B31] Meng X. Lindahl M. Hyvonen M. E. Parvinen M. de Rooij D. G. Hess M. W. (2000). Regulation of cell fate decision of undifferentiated spermatogonia by GDNF. *Science (New York, NY)* 287 1489–1493. 10.1126/science.287.5457.1489 10688798

[B32] Moore M. W. Klein R. D. Farinas I. Sauer H. Armanini M. Phillips H. (1996). Renal and neuronal abnormalities in mice lacking GDNF. *Nature* 382 76–79. 10.1038/382076a0 8657308

[B33] Nag T. C. Wadhwa S. (2001). Differential expression of syntaxin-1 and synaptophysin in the developing and adult human retina. *J. Biosci.* 26 179–191. 10.1007/BF02703642 11426054

[B34] Ogilvie J. M. Speck J. D. Lett J. M. (2000). Growth factors in combination, but not individually, rescue rd mouse photoreceptors in organ culture. *Exp. Neurol.* 161 676–685. 10.1006/exnr.1999.7291 10686086

[B35] Popsueva A. Poteryaev D. Arighi E. Meng X. Angers-Loustau A. Kaplan D. (2003). GDNF promotes tubulogenesis of GFRalpha1-expressing MDCK cells by Src-mediated phosphorylation of Met receptor tyrosine kinase. *J. Cell. Biol.* 161 119–129. 10.1083/jcb.200212174 12682085 PMC2172872

[B36] Rothermel A. Layer P. G. (2003). GDNF regulates chicken rod photoreceptor development and survival in reaggregated histotypic retinal spheres. *Invest. Ophthalmol. Vis. Sci.* 44 2221–2228. 10.1167/iovs.02-0915 12714664

[B37] Rothermel A. Volpert K. Burghardt M. Lantzsch C. Robitzki A. A. Layer P. G. (2006). Knock-down of GFRalpha4 expression by RNA interference affects the development of retinal cell types in three-dimensional histiotypic retinal spheres. *Invest. Ophthalmol. Vis. Sci.* 47 2716–2725. 10.1167/iovs.05-1472 16723491

[B38] Rowan S. Cepko C. L. (2004). Genetic analysis of the homeodomain transcription factor Chx10 in the retina using a novel multifunctional BAC transgenic mouse reporter. *Dev. Biol.* 271 388–402. 10.1016/j.ydbio.2004.03.039 15223342

[B39] Sariola H. Saarma M. (2003). Novel functions and signalling pathways for GDNF. *J. Cell. Sci.* 116(Pt 19) 3855–3862. 10.1242/jcs.00786 12953054

[B40] Schuchardt A. D’Agati V. Larsson-Blomberg L. Costantini F. Pachnis V. (1994). Defects in the kidney and enteric nervous system of mice lacking the tyrosine kinase receptor Ret. *Nature* 367 380–383. 10.1038/367380a0 8114940

[B41] Sterling P. Matthews G. (2005). Structure and function of ribbon synapses. *Trends Neurosci.* 28 20–29. 10.1016/j.tins.2004.11.009 15626493

[B42] Takahashi M. (2001). The GDNF/RET signaling pathway and human diseases. *Cytokine Growth Factor Rev.* 12 361–373. 10.1016/s1359-6101(01)00012-0 11544105

[B43] Wang N. K. Tosi J. Kasanuki J. M. Chou C. L. Kong J. Parmalee N. (2010). Transplantation of reprogrammed embryonic stem cells improves visual function in a mouse model for retinitis pigmentosa. *Transplantation* 89 911–919. 10.1097/TP.0b013e3181d45a61 20164818 PMC2855750

[B44] Wei D. Jin Z. Jarlebark L. Scarfone E. Ulfendahl M. (2007). Survival, synaptogenesis, and regeneration of adult mouse spiral ganglion neurons in vitro. *Dev. Neurobiol.* 67 108–122. 10.1002/dneu.20336 17443776

[B45] Will E. Klump H. Heffner N. Schwieger M. Schiedlmeier B. Ostertag W. (2002). Unmodified Cre recombinase crosses the membrane. *Nucleic Acids Res.* 30 e59. 10.1093/nar/gnf059 12060697 PMC117301

[B46] Wu W. C. Lai C. C. Chen S. L. Sun M. H. Xiao X. Chen T. L. (2004). GDNF gene therapy attenuates retinal ischemic injuries in rats. *Mol. Vis.* 10 93–102. 14961006

[B47] Yan Q. Wang J. Matheson C. R. Urich J. L. (1999). Glial cell line-derived neurotrophic factor (GDNF) promotes the survival of axotomized retinal ganglion cells in adult rats: comparison to and combination with brain-derived neurotrophic factor (BDNF). *J. Neurobiol.* 38 382–390. 10.1002/(sici)1097-4695(19990215)38:3<382::aid-neu7<3.0.co;2-5 10022580

