# Supplementary figures and images for: Conditional Deletion of Activating Rearranged During Transfection Receptor Tyrosine Kinase Leads to Impairment of Photoreceptor Ribbon Synapses and Disrupted Visual Function in Mice
Source: Front Neurosci. 2021 Nov 5;15:728905. doi: 10.3389/fnins.2021.728905 (PMC8602685; doi:10.3389/fnins.2021.728905)

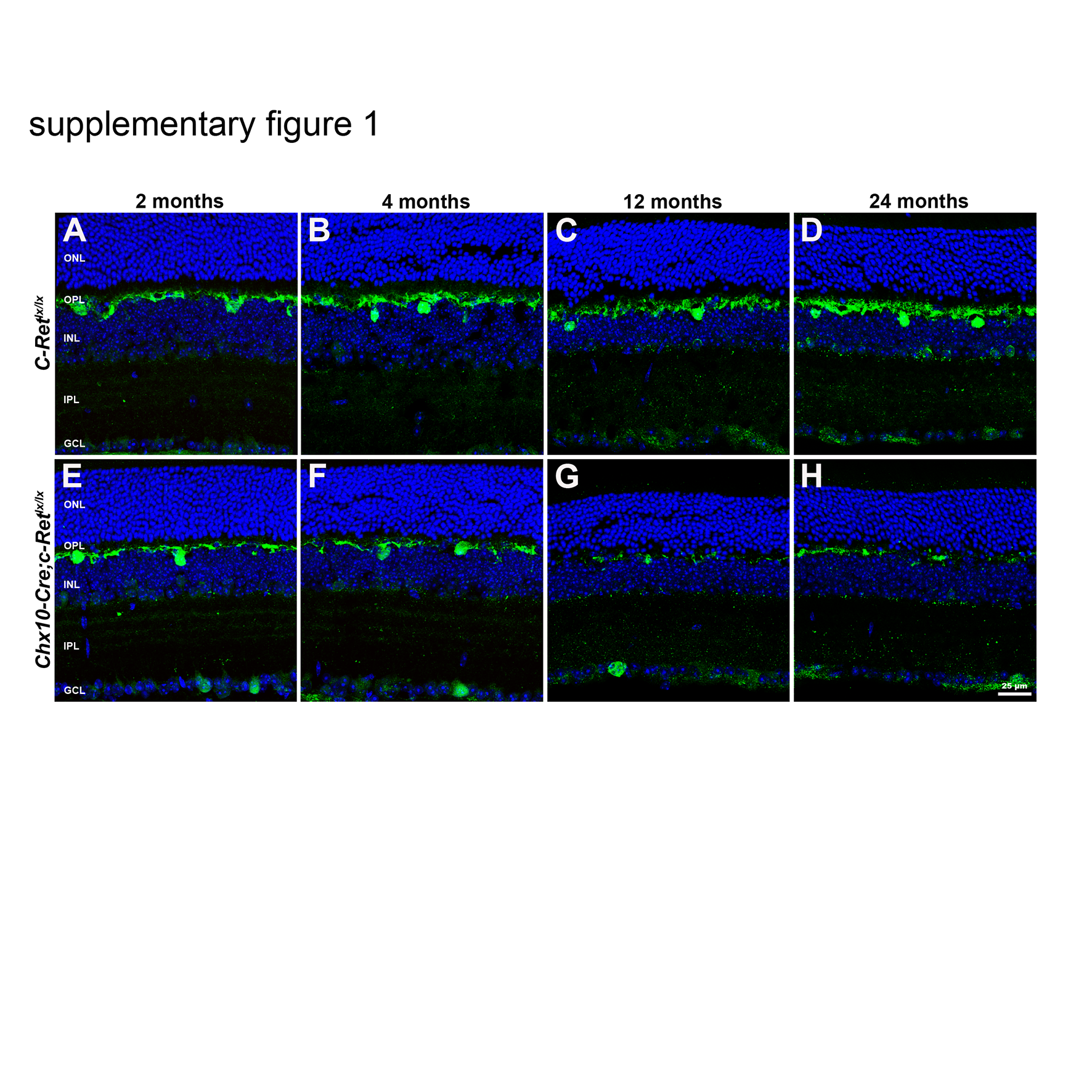

Supplement: Supplementary Figure 1 — Calbindin staining of horizontal cells in C-Retlx/lx and Chx10-Cre;C-Retlx/lx retinas. Immunostaining against calbindin (green), a specific marker for horizontal cells, followed by counterstaining with Hoechst dye to indicate cell nuclei (blue) is shown. Compared to those of C-Retlx/lx mice (A–D), confocal microscopic images of horizontal cells at 2 month (E), 4 months (F), 12 months (G), and 24 months (H) of Chx10-Cre;C-Retlx/lx mice displayed a progressed decrease in calbindin-positive immunoreactivity with an increase in age. ONL, outer nuclear layer; OPL, outer plexiform layer; INL, inner nuclear layer; IPL, inner plexiform layer; GCL, ganglion cell layer. Scale bar, 25 μm. [file Image_1.tif]

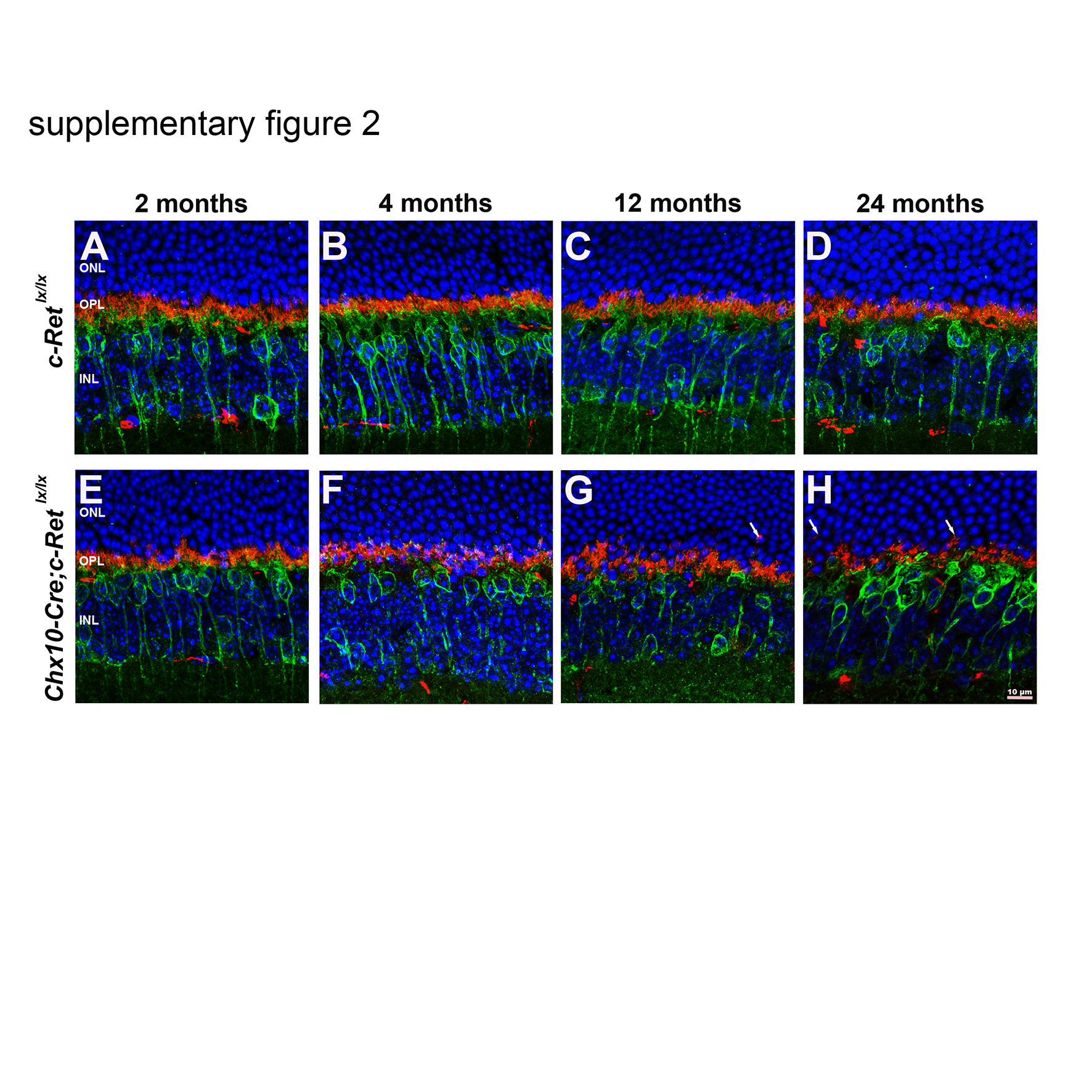

Supplement: Supplementary Figure 2 — Mislocalization of photoreceptor synaptic terminals observed in Chx10-Cre;C-Retlx/lx mice. Retinal sections of animals aged 2, 4, 12, and 24 months were immunostained with an anti-PKC-α antibody (green) and an anti-PSD95 antibody (red), to label rod bipolar cells and the synapses of photoreceptors, respectively, followed by counterstaining with Hoechst dye, to indicate cell nuclei (blue) (A–H). The processes of rod bipolar cells formed synapses with photoreceptors in the OPL of C-Retlx/lx mice at all ages (A–D) and Chx10-Cre;C-Retlx/lx mice at the ages of 2 and 4 months (E–F). However, extended rod bipolar cell dendrites and ectopic photoreceptor terminals (arrows) in the ONL were observed in 12- and 24-month-old Chx10-Cre;C-Retlx/lx mice (G,H). ONL, outer nuclear layer; OPL, outer plexiform layer; INL, inner nuclear layer. Scale bar, 10 μm. [file Image_2.tif]
